# The impact of social networking addiction on the academic achievement of university students globally: A meta-analysis

**DOI:** 10.1016/j.puhip.2025.100584

**Published:** 2025-01-13

**Authors:** Nader Salari, Hosna Zarei, Shabnam Rasoulpoor, Hooman Ghasemi, Amin Hosseinian-Far, Masoud Mohammadi

**Affiliations:** a Department of Biostatistics, School of Health, Kermanshah University of Medical Sciences, Kermanshah, Iran; b Student Research Committee, Kermanshah University of Medical Sciences, Kermanshah, Iran; c Department of Psychiatric Nursing, School of Nursing and Midwifery, Urmia University of Medical Sciences, Urmia, Iran; d Department of Business Analytics & Systems, University of Hertfordshire, Hatfield, AL10 9EU, UK; e Research Center for Social Determinants of Health, Jahrom University of Medical Sciences, Jahrom, Iran

**Keywords:** Social network, Addiction, Academic performance, University students

## Abstract

**Objective:**

There have been a significant surge in the adoption of social networks by different groups over the past decade and students are no exception. These networks create several opportunities for university students, yet they pose a number of threats. Excessive use of social networks can lead to addiction to these networks and can affect students’ academic performance. The aim of this study was to investigate the effect of social media addiction on academic performance of students around the world through a systematic review and meta-analysis.

**Study design:**

systematic review and meta-analysis.

**Methods:**

In this systematic review and meta-analysis, the keywords of Social Network, Addiction, Academic Performance, and University Students, and their suitable combinations were searched within PubMed, Web of science, JISC Library Hub Discover, the Library of congress, and the Google scholar search engine with no lower time limit and until February 2022. The identified sources were then transferred into the EndNote reference management software. Subsequently, duplicate studies were eliminated, and the remaining studies were evaluated in 3 stages (Screening, Eligibility Evaluation, and Quality Assessment). Heterogeneity of studies was assessed using the I^2^ index, analysis of eligible studies was completed be embracing the random effects model, and the data analysis was performed within the Comprehensive Meta-Analysis (v.2) software.

**Results:**

The correlation obtained from meta-analysis −0.172 (95 % CI: −0.320 - (-0.016)) showed that the negative impact of social network addiction on students' academic achievement.

**Conclusion:**

There is a negative relationship between social media addiction and students' academic performance. Therefore, it is necessary for students to be aware of the negative consequences of addiction to social networks and improve their academic performance by managing the time when using these networks.

## 1 Background

Social networks are a set of websites and applications that enable individuals and communities to connect, discuss and exchange information, and/or produce and share contents [1,2]. Today, due to the rapid advancement of technology and the typical effortless access to smartphones, the use of social networks has been growing expeditiously [3,4]. Instagram, Telegram, Facebook, Twitter, Skype and WhatsApp are some of the most popular social networks among users [5,6].

According to Ref. [7], the number of active users of social networks exceeds 3.96 billion in 2020. Research shows that among adults aged 18–29 living in the United States, social media use increased from 12 % in 2005 to 90 % in 2018 [8]. Additionally, more than 30 billion pieces of content are shared on Facebook every month, and users of this network install 20 million applications daily. On YouTube, every hour, 10 h of content is being uploaded to the video sharing platform [9].

According to Ref. [10], a big proportion of social network users are university students. They are one of the most active audiences on social networks and spend several hours online each day [11]. The use of these networks can have both positive and negative effects on students' academic performance [12]. However, based on a study by Woods et al. that the adverse effects of these networks outweigh the positive effects [13].

Social networks provide students with numerous opportunities to improve learning and access to the latest information through communication with groups and other educational systems [14]. Social networks can play a positive role in students' learning and academic performance improvement by: reducing barriers to communication and group interaction [15], supporting participatory learning activities [16], supporting active and social learning, encouraging self-study [17], increasing learning motivation [18], and increasing students' interaction with each other and with educators [19].

On the other hand, if the use of these networks is poorly managed, it can have negative consequences for students. Addiction to social networks is one of the consequences that many users of these networks may experience [20]. Addiction to social networks means excessive use of these networks and lack of control that seriously damages the lives of students [21].

Decreased academic performance is one of the most important consequences of social network addiction for students [22]. Since students tend to spend a lot of time on non-educational goals in social networks; this causes distraction from the learning environment [23,24], and can negatively affect students' academic performance by reducing the level of focus [22,[25], [26], [27]]. In this regard, studies have confirmed that excessive use of social networks causes negative effects on students' academic performance [[28], [29], [30]].

The number of social networking platforms and the number of students who use these networks are increasing day by day. This has raised concerns about social media addiction and its educational implications for students. Students use social networks for academic and non-academic purposes. Therefore, it is necessary to determine the function of these networks in the academic performance of students. The purpose of this study was to investigate the effect of social media addiction on the academic performance of students around the world. As highlighted earlier within the manuscript, this study is a systematic review and meta-analysis. The details about the adopted methodology are highlighted in next section. Subsequently, results and discussions are provided.

## 2 Methods

For this systematic review and meta-analysis, we conducted the initial search for study identification in November 2021. To do this, PubMed, Web of Science (WoS), JISC Library Hub Discover, and Library of congress databases, and the Google Scholar search engine were searched using the keywords of Social Network, Social Networking, Addiction, Academic Performance, University Students, and their pertinent combinations. These databases were selected in order to comprehensively search all databases. In order to maintain the comprehensiveness of the search, no restrictions were imposed on the language of studies, and the publication year of the articles. The identified information about articles was transferred into the EndNote reference management software. In order to maximize the number of related studies, and to identify potential grey literature, the reference lists within the identified related articles were manually reviewed. The search process was last updated in February 2022.

### 2.1 Inclusion and exclusion criteria

The criteria for including studies in the systematic review were: 1. Descriptive-analytical studies (correlation) that reported the effect of social network addiction on students' academic performance, 2. Studies that their full texts were accessible, 3. Studies that provided sufficient data (number of samples, correlation between social media addiction and academic performance).

On the contrary, the following criteria was used to exclude some of the identified articles: 1. Case control and cohort studies, 2. Case series, 3. Case reports, 4. Review studies, 5. Duplicate studies, and 6. Studies with insufficient data.

### 2.2 Study selection

**Fig. 1:**
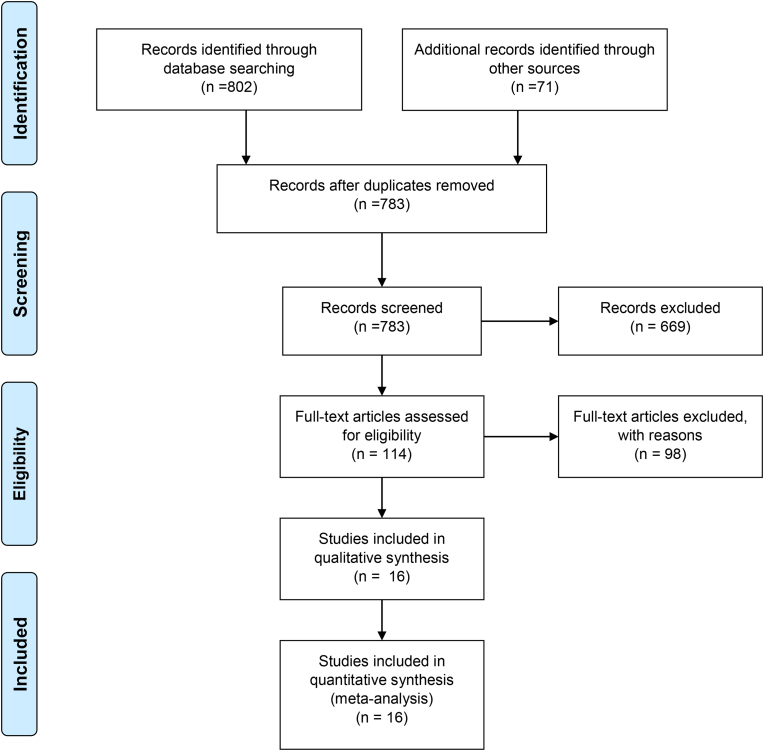
PRISMA flow diagram the meta-analysis article selection.

As highlighted earlier, the EndNote platform was used to organize the identified articles. Initially, studies that were duplicates in various searched databases were omitted from the list. As part of the initial screening, the titles and abstracts of the articles were carefully examined, and irrelevant studies were removed. In the second stage, i.e. the eligibility evaluation, the full texts of the relevant articles were reviewed, and studies that met the inclusion criteria were retained for further assessment. In order to increase credibility and prevent bias, data sources were reviewed and extracted by two independent reviewers, and in cases where there was disagreement between the two reviewers, the disputed article was assessed by a third reviewer, with a view to reaching a consensus. Finally, 16 studies entered the third stage that is quality assessment Fig. 1.

### 2.3 Quality Assessment

In order to evaluate the quality of the articles, a checklist appropriate to observational studies was adopted. The Strengthening the Reporting of Observational Studies in Epidemiology (STROBE) checklist consists of six scales that are: title, abstract, introduction, methods, results, and discussion. In total, these 6 scales contain 32 sub-scales/items. These 32 items include various methodological aspects of a study including title, problem statement, study objectives, type of study, study statistical population, sampling method, sampling strategy, definition of variables and procedures, study data collection methods, statistical analysis methods, and findings. Accordingly, articles with scores of 16 and above were considered as articles with good and average methodological quality respectively, and articles with scores below 16 were deemed as low-quality articles, and were therefore excluded.

### 2.4 Data extraction

Characteristics of final selected articles were extracted by two reviewers using a different pre-prepared checklist. This checklist included: author's name, year of publication, research location, sample size, gender and age of participants, data collection methods, and outcome.

### 2.5 Statistical analysis

The heterogeneity of studies was examined using the I^2^ test and due to the high heterogeneity, the random effects method was used to analyze the results. Additionally, the potential publication bias was tested using the Egger’s test, and corresponding funnel plots were drawn.

## 3 Results

In this systematic review and meta-analysis, we placed the focus on assessing the effect of social network addiction on students' academic performance. The study selection and review were conducted in accordance with the PRISMA protocol and guidelines. First, 802 possible related studies were identified after searching selected databases. An additional 71 articles were found through a manual search process (i.e. searching the reference lists of initially identified articles. Information about all identified articles were then transferred into the EndNote reference management software. Out of a total of 873 studies, 90 articles were duplicates and were therefore excluded. In the initial screening phase, the titles and abstracts of the remaining studies were examined, and 669 studies were excluded after considering the inclusion and exclusion criteria. In the subsequent screening stage, and after reviewing the full texts of the remaining studies, 98 further studies were omitted based on the inclusion and exclusion criteria. In the quality assessment stage, by studying the full text and based on the scores obtained from the STROBE checklist, studies that had low methodological quality were eliminated, and finally 16 studies were included in the final analysis. The characteristics of the final selected studies are provided in Fig. 1 and Table 1.

**Table 1:** Summary of selected studies' characteristics.

Author	Year	Region	Study Population	Age	Correlation Coefficient	p-value	Instruments
JJ Al Menayes.et al. [31]	2015	Kuwait	1327	18–31	r = 0.129	0.001	(SMAS), (GPA), Likret scale
K Alyafi.et al. [32]	2018	Qatar	273	N/A	r = −0.23	0.01	(CIAS), (GPA)
AM Bhandark.et al. [33]	2021	India	400	N/A	r = −0.108	0.031	(SMAS), (SPSS)
J Das.et al. [34]	2021	India	125	19–27	r = −0.527	0.005	(SNAS), (SPSS), online questionnaire
Y Li.et al. [35]	2019	China	427	N/A	r = 0.18	0 .01	(WAS), (GPA), (SPSS), Wechat use intensity scale
WAMW Pa.et al. [30]	2021	Malaysia	91	N/A	r = −0.019	0.048	(SMAS), (GPA), (SPSS), Likret scale
SM Azizi.et al. [28]	2019	Iran	360	25.48	r = −0.210	0.01	(BSMAS), (GPA), (SPSS), Likret scale
A Akalin.et al. [36]	2021	Turkey	313	21.08	r = −0.364	0.001	(SMAS-SF), (HLBS-II), (SPSS)
JV Murcia.et al. [37]	2015	USA	252	N/A	r = − 0.230	0.05	(BFAS), (SHQ)
N Sharmin.et al. [38]	2019	Bangladesh	161	21.98	r = −0.27	0.01	(BFAS), (SPSS), Independent sample *t*-test
N Dubuaku.et al. [9]	2020	Nigeria	400	18–55	r = 0.649	0.01	Google form
EL Anierobi.et al. [39]	2021	Nigeria	965	N/A	r = 0.084	0.007	(SMAS), (APS), (CGPA), Likret scale
R Glass.et al. [40]	2014	China	209	18–24	r = 0.24	0.15	(BFI), (IA-T), (GPA), Likret scale
T Koc.et al. [41]	2020	Turkey	711	N/A	r = 0.024	0.521	(GPA), (SNAIS)
A Pekpazar.et al. [42]	2021	Turkey	378	23.8	r = −0.11	0.01	(IAS), (PS), (SES), (GPA), (SPSS)
MW Tufaila.et al. [43]	2015	Pakistan	80	22–32	r = −0.896	0.01	(BFAS), (SPSS)

### 3.1 Heterogeneity, publication bias, and meta-analysis

**Fig. 2:**
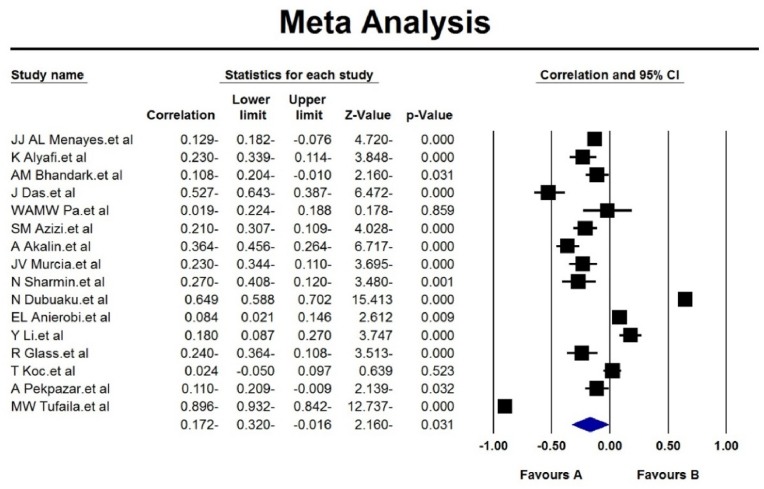
Forest plot diagram Analysis of meta-analysis results according to random effects method.

**Fig. 3:**
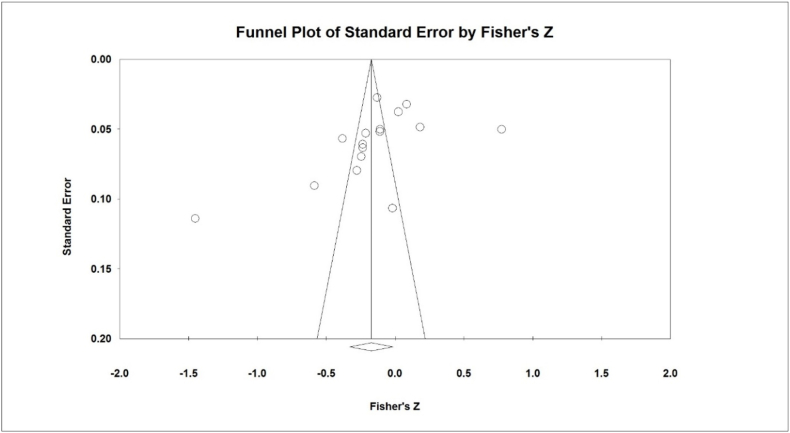
Funnel plot diagram of studies reviewed to investigate publication bias.

According to the results of the I^2^ test for assessing the heterogeneity of studies (I^2^: 97.4 %), and due to the high heterogeneity, the random effects method was used to analyze the results. The analysis of the pooled meta-analysis of 16 studies with the sample size of 6472 demonstrates the correlation of −0.172 (95 % CI: −0.320 - (-0.016), which denotes the negative impact of social media addiction on students' academic achievement (Fig. 2). Publication bias was not statistically significant (p = 0.124) (Fig. 3).

## 4 Discussion

In this meta-analysis, the correlation between social media addiction and students 'academic performance was reported to be −0.172, and our findings showed that social media addiction has a negative effect on students' academic performance.

Social networks are a group of Internet-based application networks that allow users to interact, and share information, ideas, personal messages, and other textual, visual, and audio contents [44,45]. In today's interconnected world, social networks are considered as powerful communication platforms that almost everyone uses to socialize in the cyberspace. By signing up and creating an account in these networks, individuals and groups can communicate with others immediately, regardless of their location, as long as there is an Internet connection [46].

Nowadays, social networking websites have become a global phenomenon [47]. The pervasiveness of social networks such as Twitter, Instagram and Snapchat are growing at an unprecedented rate [[48], [49], [50]]. According to reports, 4.20 billion people, or 53 % of the world's population, are active users of social networks as of 2021 [51]. Facebook, as an instance, has 1.49 billion daily active users, and this rate is increasing year by year [52].

Studies show that the largest active users of social networks are students. They spend significant amounts of time on these sites as part of their daily activities [28]. The results of a survey of 3000 students in the United States show that 90 % of students are Facebook users, and 37 % of them are Twitter users [53]. One of the applications of social networks for students is the use of these networks for academic purposes [54,55].

Social networks play a vital role in learning and teaching activities; In fact, they provide students with numerous opportunities to improve their learning. Additionally, such systems enable students to access the latest information by connecting with learning groups and other educational communities [14]. Students can also exchange information by communicating with different people; This can have a positive effect on students' learning outcomes, and academic performance [56]. In this regard, many social networking sites such as Edmodo are specifically designed for learning [57]. Additionally, Additionally, reports in Australia show that 67 % of students attribute their academic success to using technology [8].

Although social networks have several positive effects and have created opportunities to facilitate and improve the quality of education, they have also posed threats. Therefore, if students cannot manage their time when using these networks, they will face their negative consequences, including addiction [20].

Addiction to social networks denotes excessive anxiety that arises from a strong motivation to use social networks, and can essentially also mean spending a lot of time on these networks [58]. Addiction to social networks also come with adverse outcomes such as decreased academic performance [[59], [60], [61]], decreased social interactions [62], sleep deprivation [63,64], decreased level of physical activity [62], depression [65,66], and is also associated with increased anxiety [67], and other behavioral disorders [68].

Various pieces of research on social media addiction show the prevalence of social network addiction in the student population is 59.4 % and such addiction can occur to anyone regardless of age, gender, or education [[69], [70], [71], [72], [73]]. However, students are more vulnerable to social media addiction for a variety of reasons; First, university students have a high level of Internet literacy, which allows them to be the main users of social networks [74,75]. Second, parents have less control over university students' online activities than high school students. Third, students have more flexible schedules and unlimited access to social networks. Fourth, youth-related personality traits may increase the attractiveness of social media for students [76].

Academic performance has various definitions within the literature, though it typically refers to the “overall performance in each year leading to the GPA” [77]. Decreased academic performance is one of the most important consequences of social media addiction for students. In this regard, researchers in a study showed that addiction to social networks hurts academic achievement by creating academic procrastination and increasing academic stress [25]. The students who used social networks too much had poorer academic achievement and lower concentration in the classrooms [22]. The results of another study on a group of students in Qatar demonstrate that the average score (GPA) among university students addicted to social networks is lower than other students [32].

It has been reported that social media addiction and academic performance are linked to personality [73,78,79]. Extroverts, due to their desire to socialize with others, see social media as a suitable environment to express themselves and meet their needs and wants [[80], [81], [82], [83], [84], [85], [86], [87]]. This results in them spending significant amounts of time on social networks and becoming addicted to these networks. Introverts, on the other hand, may compensate for their lack of social relationships through the overuse of social networks [88].

Individuals with neuroticism suffer from feelings of instability and impatience, and can get angry quickly [89]. These people may spend many hours on social media to escape their troubled social relationships in real life. This causes this group of people to become addicted to social networks [90]. Other studies have shown that social media addiction is negatively affected by the neurotic dimension; Accordingly, neurotic people are less addicted to social networks since these individuals become nervous quickly, and do not prefer to have routine activities [[91], [92], [93], [94], [95], [96], [97], [98], [99], [100], [101], [102], [103], [104], [105], [106], [107], [108], [109], [110], [111]].

Conscientious individuals avoid any kind of addiction due to personality traits such as responsibility, discipline, planning, hard work, punctuality, reliability, and perseverance. In other words, for addiction to social networks, a lot of time should be devoted to the use of these networks, which is contrary to the conscientious characteristics of individuals [[112], [113], [114], [115], [116], [117], [118], [119], [120], [121]]. Nevertheless, some research shows that conscientiousness plays an important role in the formation of social network addiction [[122], [123], [124], [125], [126], [127], [128], [129], [130], [131], [132], [133]].

Social media addiction is positively affected by the open dimension [[83], [84], [85], [86]]; Openness means embracing the experience of new and non-traditional ideas, the search for novelty, diversity, and intelligent, imaginative, and intellectual curiosity. People with an open personality tend to try new things, and social media always contains new and exciting ideas that come from new cultures, motivations, and events. Therefore, people with openness tend to have a high tendency to use social networks and are prone to addiction to social networks [83,86,[93], [94], [95]]. In contrast, some studies show that openness is not associated with the use of social networks [81,84].

## 5 Strengths and limitations

Among the most important strengths of this study is the comprehensive review of all review databases and the meta-analysis of the correlations presented by these articles. The most important limitation of the present study was the lack of sufficient information in the reviewed articles to perform subgroup analysis.

## 6 Implications

The findings of this study show the negative effect of addiction to social networks on the academic progress of students and such results can be taken into consideration by teachers and parents of students in examining the academic performance of students as well as examining their academic failures. Therefore, in addition to making students aware of the negative impact of addiction to social networks, students prone to addiction to social networks can be identified and followed up.

## 7 Conclusion

Our findings demonstrate that social media addiction negatively affects university students' academic performance. Given the negative effects of social networks on students' academic performance, the issue of social media addiction should be considered by students, educators, and policymakers. In this regard, it is necessary to consider further research priorities to assess the applications of social networks at the individual and social levels of among university students.

## Ethics approval and consent to participate

Ethics approval was received from the ethics committee of deputy of research and technology, Kermanshah University of Medical Sciences (50001746).

## Consent for publication

Not applicable.

## Availability of data and materials

Datasets are available through the corresponding author upon reasonable request.

## Authors'contributions

MM and NS and SHR contributed to the design, MM statistical analysis, participated in most of the study steps. SHR and HZ and AHF and MM and HGH prepared the manuscript. All authors have read and approved the content of the manuscript.

## Funding

By Deputy for Research and Technology, Kermanshah University of Medical Sciences (IR) (50001746). This deputy has no role in the study process.

## Declaration of competing interest

The authors declare that they have no known competing financial interests or personal relationships that could have appeared to influence the work reported in this paper.

## Abbreviations

WoS: Web of Science
STROBE: Strengthening the Reporting of Observational studies in Epidemiology
PRISMA: Preferred Reporting Items for Systematic Reviews and Meta-Analysis
